# *Cryptococcus neoformans*-Derived Microvesicles Enhance the Pathogenesis of Fungal Brain Infection

**DOI:** 10.1371/journal.pone.0048570

**Published:** 2012-11-07

**Authors:** Sheng-He Huang, Chun-Hua Wu, Yun C. Chang, Kyung J. Kwon-Chung, Robert J. Brown, Ambrose Jong

**Affiliations:** 1 Division of Infectious Diseases, The Saban Research Institute, Children’s Hospital Los Angeles, Department of Pediatrics, Keck School of Medicine, University of Southern California, Los Angeles, California, United States of America; 2 Division of Hematology-Oncology, The Saban Research Institute, Children’s Hospital Los Angeles, Department of Pediatrics, Keck School of Medicine, University of Southern California, Los Angeles, California, United States of America; 3 Laboratory of Clinical Infectious Diseases, National Institute of Allergy and Infectious Diseases, Bethesda, Maryland, United States of America; University of Minnesota, United States of America

## Abstract

Cryptococcal meningoencephalitis is the most common fungal disease in the central nervous system. The mechanisms by which *Cryptococcus neoformans* invades the brain are largely unknown. In this study, we found that *C. neoformans*-derived microvesicles (CnMVs) can enhance the traversal of the blood-brain barrier (BBB) by *C. neoformans in* *vitro*. The immunofluorescence imaging demonstrates that CnMVs can fuse with human brain microvascular endothelial cells (HBMECs), the constituents of the BBB. This activity is presumably due to the ability of the CnMVs to activate HBMEC membrane rafts and induce cell fusogenic activity. CnMVs also enhanced *C. neoformans* infection of the brain, found in both infected brains and cerebrospinal fluid. In infected mouse brains, CnMVs are distributed inside and around *C. neoformans*-induced cystic lesions. GFAP (glial fibrillary acidic protein)-positive astrocytes were found surrounding the cystic lesions, overlapping with the 14-3-3-GFP (14-3-3-green fluorescence protein fusion) signals. Substantial changes could be observed in areas that have a high density of CnMV staining. This is the first demonstration that *C. neoformans*-derived microvesicles can facilitate cryptococcal traversal across the BBB and accumulate at lesion sites of *C. neoformans*-infected brains. Results of this study suggested that CnMVs play an important role in the pathogenesis of cryptococcal meningoencephalitis.

## Introduction

Cryptococcal meningoencephalitis is the most common fungal disease in the central nervous system. It is also a major cause of death in AIDS patients. To infect the brain, *C. neoformans* must cross the blood-brain barrier (BBB). The BBB is formed mainly by brain microvascular endothelial cells (BMECs) with tight junctions between the cells [1]. Using human BMECs (HBMECs) as an *in* *vitro* model of BBB, we have previously found that *C. neoformans* crosses the BBB through a zipper mechanism [2], and CD44 on the lipid rafts may play the role as the host receptor for *C. neoformans* invasion [3]–[5]. However, the definitive mechanisms of *C. neoformans* brain invasion and infection are still largely unknown.

*C. neoformans* is enveloped by a polysaccharide capsule which is responsible for a number of deleterious effects, including interference with phagocytosis, modulation of cytokine production, and inhibition of leukocyte migration [6]. The capsule is composed primarily of glucuronoxylomannan and other polysaccharide derivatives. Since acapsular mutants are avirulent, the *C. neoformans* capsule is considered as a virulence factor [7]. *C. neoformans* produces extracellular microvesicles (CnMVs) during *in* *vitro* growth and animal infection, and these vesicular compartments contain capsule components [8], [9], suggesting that these vesicles are the carriers for extracellular transport of this polysaccharide. As such, vesicles traverse across the cell wall to release building blocks for capsular biosynthesis.

Recently, it has been discovered that a number of pathogens produce extracellular microvesicles (MVs) that cause disease [10]; such as the eukaryotic parasites *Leishmania* [11], [12] and gram-negative bacteria *Legionella* [13]. Their MVs have been found to contain various components associated with their virulence. Moreover, other microbial pathogens, such as *H. pylori*, *A. actinomycetemcomitans*, *Salmonella typhimurium*, *Bacteriodes gingivalis*, and the *Bacteriodes* species, also secrete MVs. These released MVs are known to play important roles in gastric ulcers, periodontitis, and other similar diseases (reviewed in [14]). Moreover, viruses and prions utilize the host-secreted MVs to infect neighboring cells [15], . Thus, these pathogenic MVs expand the current paradigms of pathogen invasion. Accordingly, further investigation of the roles of extracellular MVs may lead to exciting new developments in infectious disease research.

The production and secretion of CnMVs are normal processes of *C. neoformans* as its secretion mutant *sav1* (a homolog of Sec4/Rab8 GTPase), which accumulate vesicles intracellularly at 30°C, displays a reduced growth rate [17]. Experiments with another mutant, *sec6*, have demonstrated the association between vesicle secretion and pathogenesis of *C. neoformans* [18]. Several secretion-related genes such as *snf7*, *sec1*, *bas1,* and *vps23* are also found to be involved in the formation of microvesicles [19].

CnMVs also transport key lipids (e.g., glucosylceramide and sterols) and cytoplasmic proteins to the extracellular space [8]. CnMVs contain concentrated fungal products that may enhance invasion of host cells and tissue; therefore, they are thought to function as a virulent bag [20]. CnMVs have also been shown to activate antimicrobial activity of phagocytic cells [21]. These findings suggest that the secreted CnMVs cross the cell wall to enter the extracellular space not only to provide polysaccharides for capsule assembly but also to deliver various factors to host tissues.

Although CnMVs may be involved in the cryptococcal pathogenesis, their roles in the initial steps of *C. neoformans* infection especially the brain invasion are unknown. In this study, we found that purified CnMVs enhanced the traversal of *C. neoformans* across the BBB. In the infected mouse brain, CnMVs were distributed inside and around *C. neoformans*-induced cystic lesions. Substantial changes could be observed in areas with a high density of extracellular MV staining. Altogether, our study is the first demonstration of how *C. neoformans*-derived MV can facilitate traversal of the pathogen across the BBB and accumulate at the lesion sites in infected brains. The involvement of CnMVs in these processes suggests a novel mechanism of brain invasion by this pathogen.

## Results

### Construction of CnMV Marker 14-3-3-GFP Fusion

**Figure 1:**
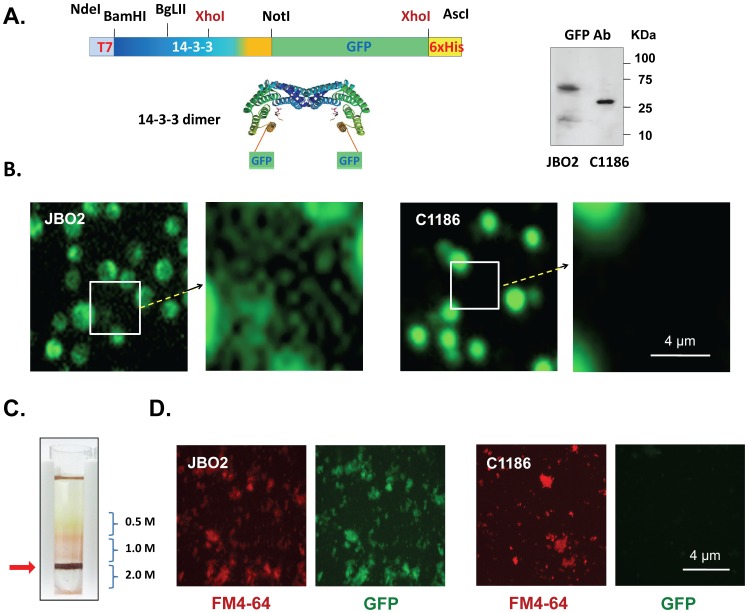
Expression of *C. neoformans* 14-3-3-GFP fusion protein and its use as a CnMV marker. The gene encoding the *C. neoformans* 14-3-3-GFP fusion protein was inserted between NdeI and AscI sites on the pGEM-MV11G (see Experimental Procedure) with a T7 tag and a 6x His tag linked at its 5′- and 3′-end, respectively. This fusion gene was driven by the actin-1 promoter. The N-terminus of 14-3-3 is marked in blue (dimerization region), and a putative random coiled region at its C-terminus is marked in gold. The schematic view of the 14-3-3 dimer shows that the GFP portion is linked to the random coiled region of its C-terminus. Cell extracts from the JB02 strain or the C1186 strain were probed with an anti-GFP antibody to detect the expressed 14-3-3-GFP and GFP. (**B**) The fluorescence images were that of the *C. neoformans* JB02 (expression of 14-3-3-GFP) and C1186 (expression of GFP only) cells in culture. Boxed regions in the JB02 and C1186 images were magnified and shown on the right side. (**C**) The location of the FM4-64 stained CnMVs from a sucrose gradient is shown (red arrow). (**D**) Aliquots of the purified CnMVs from JB02 (upper panels) or C1186 (lower panel) cells were diluted and examined by fluorescence microscopy. Bar: 4 µm.

Since proteomic studies revealed that the *C. neoformans* 14-3-3 protein is one of the most abundant proteins in CnMVs [20], it was used as a marker for CnMVs in this study. The 14-3-3 protein is a dimer, with two molecules joined together at their N-termini (Fig. 1A, blue region). A random coil structure is located at the C-terminus of the protein (Fig. 1A, gold region). To enable direct visualization of CnMVs, the green fluorescence protein (GFP) was fused to the C-terminus of 14-3-3. The expression of the 14-3-3-GFP fusion gene was driven by the actin-1 promoter, and the construct containing the fusion gene was introduced into *C. neoformans* strain B-4500FO2 to yield strain JB02. The control strain C1186 harbored the plasmid containing only the GFP gene (*i.e*., GFP was not fused to 14-3-3). The expressions of 14-3-3-GFP in JB02 and plain GFP in C1186 cells were confirmed by Western blotting using the anti-GFP antibody (Fig. 1A).

Fluorescence microscopy was then performed to detect the 14-3-3-GFP in JB02 and the plain GFP in C1186 cells. A speckled distribution pattern of the 14-3-3-GFP was observed in JB02 cells (Fig. 1B). Many small green particles surrounding JB02 cells were also observed, suggesting that some 14-3-3-GFP proteins were packed into CnMVs that were then secreted into the culture medium. In contrast, C1186 cells showed a solid bright, shiny green staining evenly distributed throughout the cells with no extracellular staining, indicating that the expressed GFP was located inside *C. neoformans* cells but not in secreted CnMVs.

To confirm the presence of CnMVs, the small particles in the culture supernatants of both JB02 and C1186 cells were pelleted by ultracentrifugation, stained with the lipophilic dye FM4-64, and then further fractionated by sucrose gradient centrifugation. Highly purified CnMVs were obtained in the top fraction of the 2.0 M sucrose cushion (Fig. 1C, red arrow). This fraction had both laccase activity and acid phosphatase activity (data not shown), as previously described in the CnMV fractions [20]. Under fluorescence microscopy, red staining (FM4-64) was observed in the CnMVs from both JB02 and C1186 strains (Fig. 1D), but green staining was only seen in JB02 CnMVs (which contained 14-3-3-GFP). This is further evidence that the 14-3-3-GFP was present in JB02 CnMVs, but the plain GFP protein from the C1186 strain was not packed into the secreted CnMVs. Overall, these data indicated that 14-3-3-GFP could be used as a specific marker for CnMVs. This study also provides a purification scheme for our CnMV studies.

### Purified CnMVs Enhanced the Traversal of *C. neoformans* Cells Across BBB *in* *vitro*

**Figure 2:**
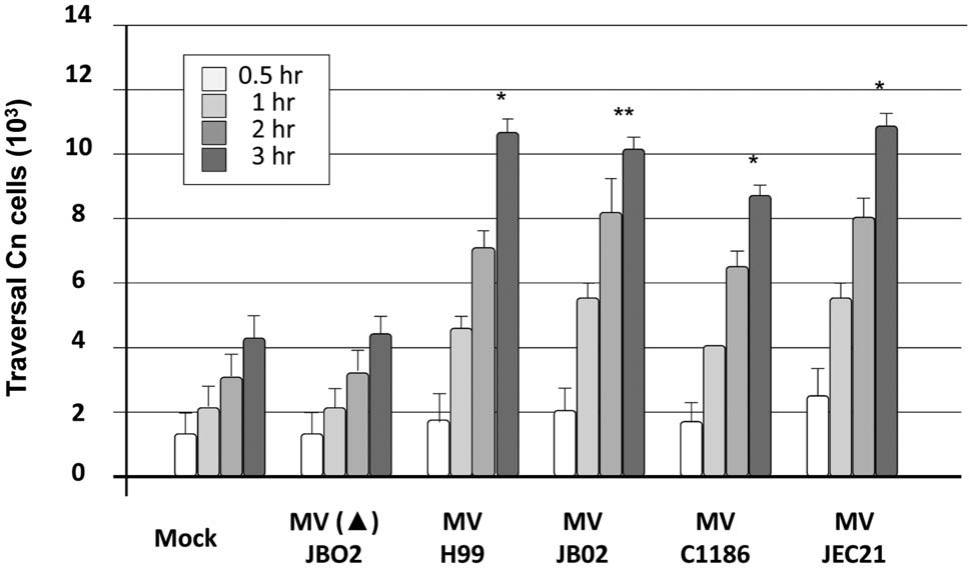
Effect of CnMVs on the traversal of *C. neoformans* cells through an HBMEC layer *in vitro*. CnMVs were isolated from different strains, as indicated on the bottom of the figure. *In vitro* transcytosis assays were performed using strain B-4500FO2 (10^6^ cells) and 10 µg purified CnMVs. No MV treatment (Mock) and treatment with heat-inactivated MVs (MV▴) from the JB02 strain were used as controls. Each group contained four bars representing the 0.5, 1, 2, and 3 hour time points. Triplicate samples at each time point were analyzed. CFUs were determined from the cultures in the lower chambers of the transwells, and the recovery percentage was determined at indicated time points (n = 3). Analysis of variance showed a significant difference between the CnMV-treated samples and the controls (mock or heat-inactivated MV) as indicated by asterisks (*P<0.05; **P<0.01).

To determine whether CnMVs from different strains of *C. neoformans* had any effect on *C. neoformans* invasion, CnMVs isolated from strains JB02, H99, C1186, and JEC21 were used to test their the ability of *C. neoformans* to traverse the BBB *in* *vitro*. As shown in Fig. 2, CnMVs purified from all the strains enhanced the crossing of the HBMEC monolayer *by C. neoformans* cells (strain B-4500FO2) *in* *vitro*. Heat-treated CnMVs [MV(▴)] lost their enhanced activity (Fig. 2, 2^nd^ group), suggesting that a heat-labile molecule(s) was involved in this activity. The trans-endothelial electrical resistances (TEER) were maintained ∼300 μΩ per cm^2^ throughout the studies; therefore, CnMVs exert no or little effects on the tight junctions of the HBMEC monolayer during the studies. Taken together, these data indicated that CnMVs can enhance the invasion of *C. neoformans* across the BBB.

### Interaction between CnMVs and HBMECs

**Figure 3:**
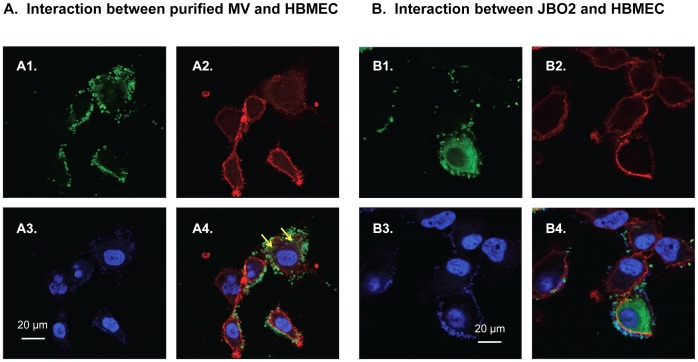
Interaction between CnMVs and HBMECs. (**A**) HBMECs were incubated with purified CnMVs and then examined by confocal microscopy. The HBMECs were stained with FITC-conjugated anti-GFP (A1), rhodamine-conjugated anti-CD44 (A2), and DAPI (A3). An overlaid image of A1, A2 and A3 is shown in A4. The yellow arrows indicate faint, diffuse staining in the cytoplasm of HBMECs. Bar: 20 µm. (**B**) HBMECs were incubated with JB02 *C. neoformans* cells and then examined by confocal microscopy. The HBMECs were stained with FITC-conjugated anti-GFP (B1), rhodamine-conjugated anti-CD44 (B2), and DAPI (B3). An overlaid image of B1, B2 and B3 is shown in B4. Bar: 20 µm.

Confocal microscopy was then performed to observe the interactions between CnMVs and HBMECs. The HBMECs were stained with a rhodamine conjugated-CD44 antibody (red), and their nuclear DNA was stained with DAPI (blue). In the presence of purified CnMVs, CD44 staining of HBMECs showed rough membrane morphology, indicating that membrane ruffling was induced. As seen in Figure 3A, the purified CnMVs (green dots) were found attached to the surface of HBMECs, but were not colocalized with CD44 (red staining). Faint, diffuse green signals were observed inside the cytosol between the plasma membrane (red) and the nucleus (blue), suggesting that some CnMVs were internalized into the cytoplasm of HBMECs (Fig. 3A, in A4 panel - yellow arrows). This intracellular accumulation was increased over time (data not shown).

When *C. neoformans* JB02 cells were incubated with HBMECs, the green CnMVs were also found attached to the surface of HBMECs. Interestingly, some HBMEC cells showed intracellular green signals (Fig. 3B, the lower cell in B1 and B4 panels), suggesting that *C. neoformans* JB02 cells had invaded the HBMECs and secreted CnMVs internally. It was difficult to distinguish between a *C. neoformans* cell and CnMVs in this image because both the JB02 cell and secreted CnMVs were stained green. Under the same experimental conditions, the C1186 strain did not show any green MV staining (data not shown). Overall, these data indicated that CnMVs from the JB02 strain could be detected on the surface of and inside the host cells.

### CnMVs Induced Redistribution of Membrane Lipid Raft Components and Cell Fusion Activity of HBMECs

To explore the mechanism how the CnMVs exert the effects on the HBMEC surface membrane, density gradient centrifugation was performed to fractionate and examine membrane lipid rafts and other cellular components. HBMECs were treated with purified CnMVs (0, 0.5, 1, or 4 µg) and lysed in a buffer containing 1% Triton X-100. The fractionation was performed in OptiPrep™ density gradients. After centrifugation, the loading buffer floated to the top (Fraction 1); the plasma membrane and the lipid rafts were in Fraction 2 as expected [5]. The next few fractions contained soluble proteins (Fractions 3–6) and cytoskeleton-associated, detergent-insoluble proteins (Fractions 7–9).

**Figure 4:**
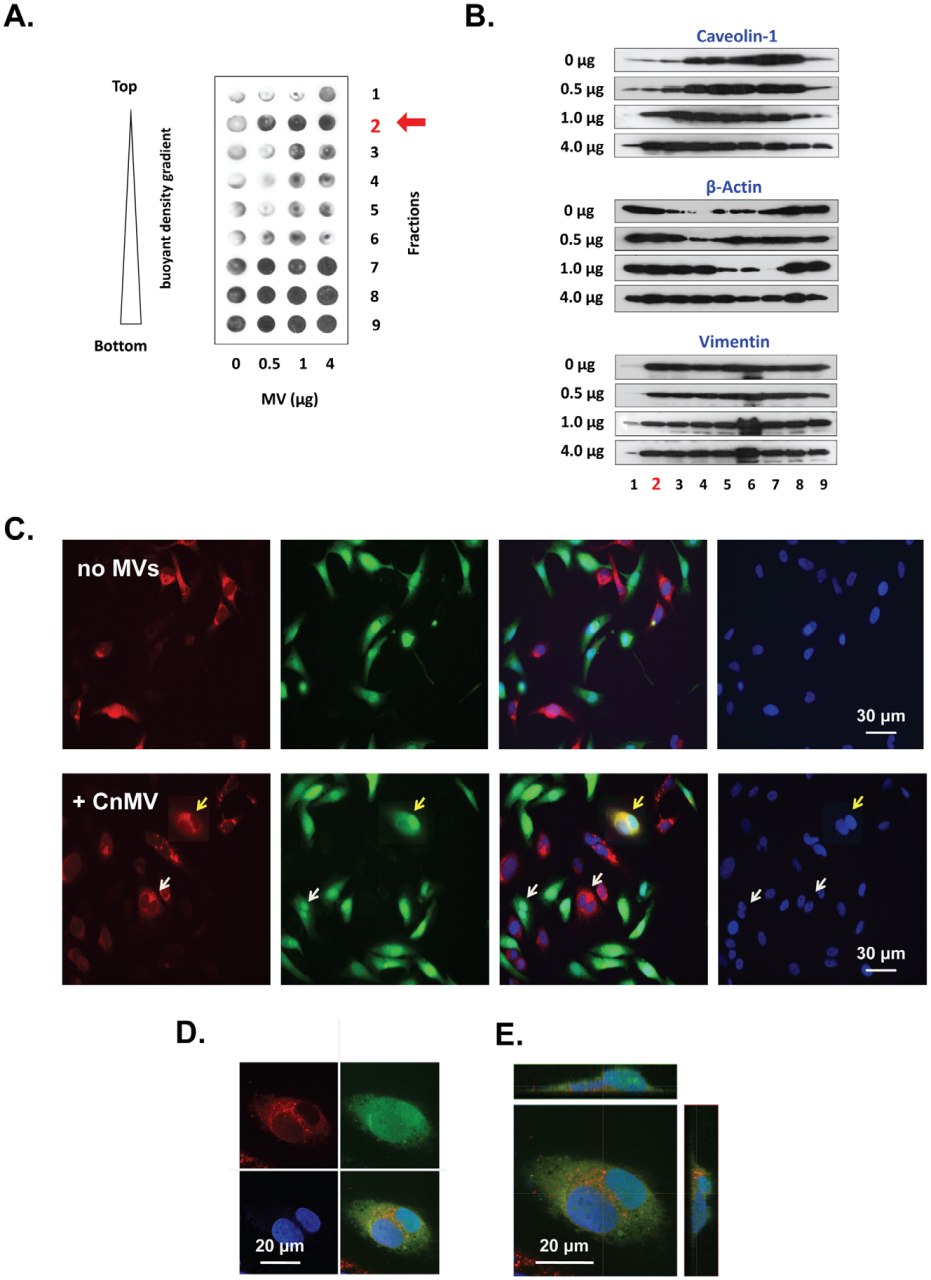
Effect of CnMVs on lipid raft activation and fusion of HBMECs. HBMECs were treated with PBS or purified CnMVs (0.5, 1, or 4 µg) for 2 h. The cells were lysed in a buffer containing 1% Triton X-100 on ice, and the cell lysates were fractionated in Optiprep™ gradients. Nine fractions were collected. The lipid raft fractions were located in Fraction 2, indicated by a red arrow. An equal volume of each sample was analyzed by dot blots with antibody against CD44 (**A**) or protein blots with antibodies against caveolin-1, β-actin, and vimentin (**B**). For cell fusion studies, D54GM cells were treated with the Celltracker red dye CMTPX (1^st^ panel) or the green dye CMFDA (2^nd^ panel) and then incubated with 10 µg purified CnMVs. Syncytial cells were detected after 6 hours of co-culture and displayed a bright yellow signal (yellow arrow) (**C**). Images shown in (**D**) are a confocal microscopic section with split images: CMFDA dye (green), CMTPX dye (red), DPAI (blue), and the overlaid image (**D**). Images shown in (**E**) are a confocal image with a top and two side views.

CD44 is the primary receptor of *C. neoformans* on HBMEC [3]–[5]. Dot blot analysis was first performed to examine the presence of CD44 in each fraction (Fig. 4A). Without the addition of CnMVs, the lipid raft fraction (Fraction 2, red arrow) of HBMECs contained very small amounts of CD44. However, the amounts of CD44 in the same fraction were increased proportionally to varying amounts of MVs added to the HBMECs. CD44 was also present in high levels in the cytoskeleton rich fractions (Fractions 7–9) of HBMECs with a slight increase in the presence of CnMVs.

**Figure 5:**
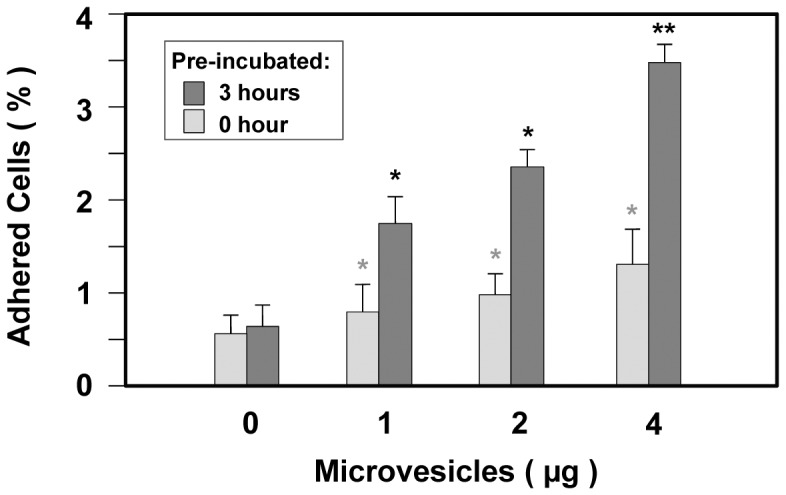
CnMVs enhanced the adhesion of *C. neoformans* cells to HBMECs. *In vitro* adhesion assays were performed in 24-well tissue plates with (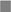) or without (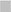) 3 hours of pre-incubation with purified CnMVs (0, 1, 2, or 4 µg) with triplicate samples in each measurements and three experiments was performed (n = 3). Analysis of variance showed that CnMV treatment led to a significant increase in adhesion (*P<0.05; **P<0.01).

Both caveolin-1 and β-actin are required in *C. neoformans* invasion [3]–[5], and they are often found on the HBMEC membrane. Therefore, caveolin-1 and β-actin levels in each fraction were then determined. The levels of vimentin were also measured to serve as the control. Both caveolin-1 and β-actin in each fraction were altered in proportion to the amounts of CnMVs added (Fig. 4B, central two panels). However, the distribution patterns of caveolin-1 and β-actin were quite different. Caveolin-1 increases more significantly in the lipid raft fraction (Fraction 2), whereas the distribution of β-actin is upshifted to the lower density fractions. These results indicated that isolated CnMVs had a profound effect on the redistribution of caveolin-1 and β-actin. In contrast, CnMVs had little effect on the distribution of vimentin (Fig. 4B, last panel). Since CD44, caveolin-1, and β-actin are involved in cell-cell interaction, cell adhesion, or migration, these results suggested that CnMVs can activate surface membrane of HBMECs and facilitate *C. neoformans* adhesion (Fig. 5) and transcytosis (Fig. 2).

Intracellular vesicles and exosomes regularly fuse to the endocytic membrane systems and plasma membrane prior to their secretions, and this fusion activity may assist in the adherence and internalization of *C. neoformans* into recipient cells. To further explore the interaction between purified CnMVs and host cells, the possibility that CnMVs could induce cell fusion was investigated. Two distinct fluorescent cytoplasmic probes, CMTPX (red) and CMTDA (green), were used to verify cytoplasmic exchanges between human cells (Fig. 4C). Host cells were split into two aliquots. Each aliquot was stained with one of the two dyes for 24 hours, and a co-culture of the two aliquots was performed in the presence of purified CnMVs from strain C1186. Within 6 hours, double-fluorescent cells were able to be detected by fluorescent microscopy in a ratio of about 1∶5,000 cells (Fig. 4C), as evidenced by the bright yellow cells in the co-culture (Fig. 4C, yellow arrow). In the absence of CnMVs, no cell fusions were observed (Fig. 4C, upper panel). As CnMVs could not discriminate between the red- and green-stained cells, a red cell fused with another red cell or a green cell fused with another green cell (Fig. 4C, white arrows in lower panel) was also observed. To rule out the possibility that the colocalized signals were due to overlapping cells, cells in the co-culture were examined by confocal microscopy, and a fused cell was scanned from top to bottom in 1-µm sections. As seen in Fig. 4D, a central section of a fused cell with binuclei displayed both green and red stains throughout the cytosol (Fig. 4D, upper panels). The overlaid image (Fig. 4D, lower right panel and Fig. 4E) of both top and side views showed yellow staining in the cytosol surrounding the binuclei. Taken together, these results demonstrated that purified CnMVs contained component(s) capable of inducing host cell fusion.

### CnMVs Enhanced *C. neoformans* Adherence to HBMECs *in* *vitro*

Experiments were then performed to investigate whether purified CnMVs have any effects on the adhesion between *C. neoformans* and HBMECs. Different amounts of purified CnMVs (0, 1, 2, or 4 µg) were added to HBMEC culture. *C. neoformans* cells were then added to the culture, and the adherence of *C. neoformans* to HBMECs was determined immediately or 3 hours after pre-incubation with CnMVs. As shown in Fig. 5, the degree of *C. neoformans* adhesion to HMBECs was CnMV dose-dependent for both sets of cultures. However, the 3-hour preincubation with CnMVs greatly intensified the adhesion.

### CnMVs Increased *C. neoformans* Load in the Brain of Infected Mice

**Figure 6:**
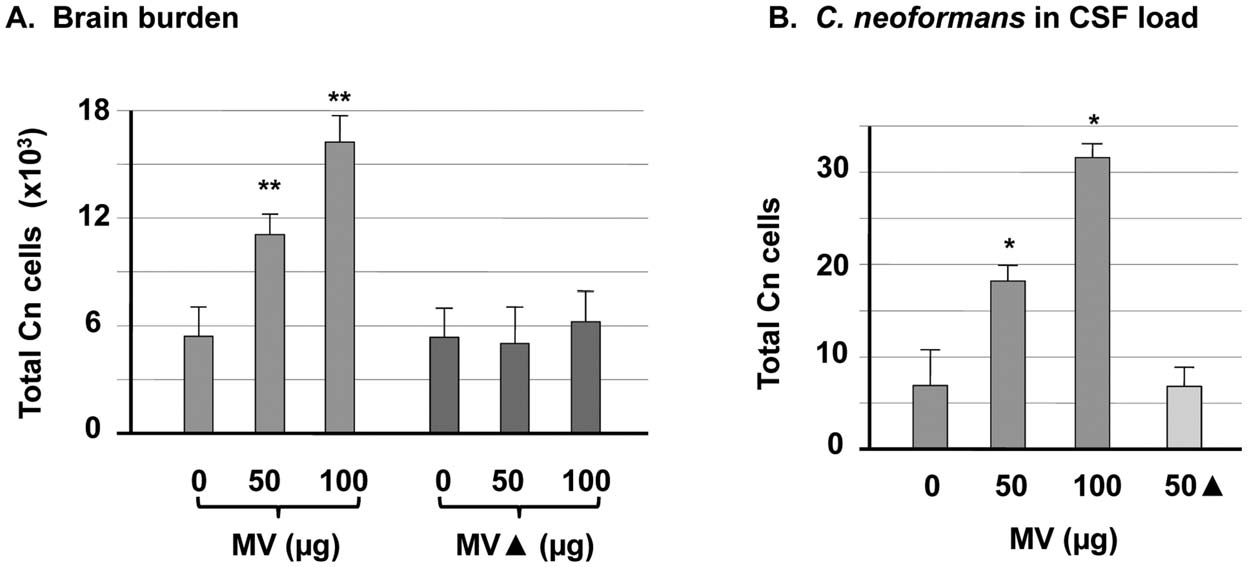
CnMVs enhanced *C. neoformans* brain infection. (**A**) Groups of 3-5 C57LB/6 mice were intravenously injected with 50–100 µg purified MVs or MV▴ (MVs heat-treated at 95°C for 10 min) and 10^6^ B-4500FO2 *C. neoformans* cells. The brains were harvested 24 hours after the injection. Quantitative cultures were performed by plating the brain homogenates on YPD medium (n = 4) (P<0.01). (**B**) The same approach as in (A) was followed except that the *C. neoformans* cells were collected from the CSF (n = 3). Data, expressed as CFU, were the means ± SD of plates inoculated in triplicate from three independent experiments (P<0.05).

Hematologically disseminated *C. neoformans* cells may secret CnMVs continuously into the blood. To explore the effect of CnMVs on *C. neoformans* invasion *in* *vivo*, purified CnMVs and *C. neoformans* cells were delivered into mice by tail vein injection. The cryptococcal brain burden was examined 24 hours after infection. Heat-treated CnMVs (MV▴) were used in an identical manner as the control. As shown in Fig. 6A, CnMVs increased the *C. neoformans* brain burden in a dose-dependent manner (P<0.01), and heat treatment of CnMVs inactivated this activity.

As the presence of *C. neoformans* in the cerebrospinal fluid (CSF) is an indication of cryptococcal meningitis, *C. neoformans* loads in CSF were determined (P<0.05; Fig. 6B). Similar to that in the brain, the number of *C. neoformans* cells in the CSF increased proportional to the amount of injected CnMVs. Moreover, heat-treated CnMVs failed to boost the number of *C. neoformans* cells in the CSF. These *in* *vivo* results were consistent with those of the *in* *vitro* studies (Figs. 2 & 5) and demonstrated that CnMVs play a significant role in *C. neoformans* brain invasion.

### Distribution of CnMVs (14-3-3-GFP) in Infected Mouse Brains

**Figure 7:**
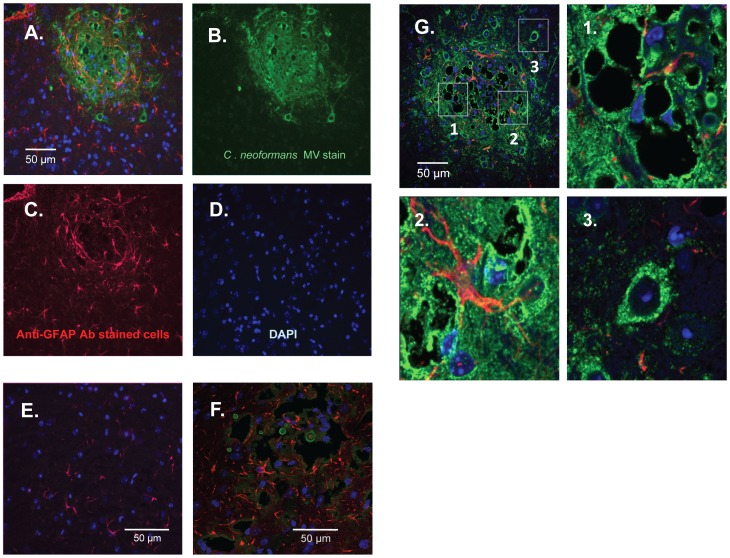
Distribution of the CnMV marker and glial cells around *C. neoformans*-induced cystic lesion in infected brains. C57LB/6 mice were infected with 10^6^ *C. neoformans* JB02 cells for 10 days. Brain sections were stained with an anti-GFP antibody to detect the CnMV marker 14-3-3-GFP (green) (**B**), an anti-GFAP (glial fibrillary acidic protein) antibody to detect host astrocyte cells (red) (**C**), and DAPI for the localization of brain cells (blue) (**D**). Image (**A**) is the overlaid image of (B, C, D). Image (**E**) is a section of an uninfected brain with no detectable staining for GFP. The *C. neoformans* strain C1186 was used to infect mice for comparison; green yeast cells were evident but CnMVs could not be detected in brain sections (**F**). A confocal image of a typical cystic lesion after 16 days of infection is shown in (**G**). Enlarged regions (box 1, 2, and 3) in image (**G**) are displayed in (**1**), (**2**)**,** and (**3**), and their corresponding videos are provided in Supplement V(1), V(2), and V(3), respectively. Bar = 50 µm.

To investigate CnMVs during infection, immunofluorescence microscopy was performed to locate CnMVs in the brain. Strain JB02 was used for the infection, and CnMVs were identified as 14-3-3-GFP (Fig. 7A & B). While intense green fluorescence was observed in the infected brain (Fig. 7A & B), it was not seen in an uninfected brain (Fig. 7E). The intensity of the 14-3-3-GFP signal increased over time (Supplement Fig. S1). At 10 days post-infection, large cystic lesions surrounded by bright green staining were observed, indicating the presence of 14-3-3-GFP (or CnMV components) in these regions (Fig. 7A and 7B). When an anti-GFAP antibody was used, GFAP-positive astrocytes were found surrounding the cystic lesions (Fig. 7C). This GFAP distribution pattern corresponded to that of 14-3-3-GFP (Fig. 7A). In uninfected brains, there was a much weaker GFAP-positive cell staining (Fig. 7E). When the C1186 strain was used, we could not detect green CnMVs (14-3-3-GFP) but observe some green *C. neoformans* cells (Fig. 7F). Although it is hard to determine whether 14-3-3-GFP was inside in an intact CnMV (Figure 7A & B), these images demonstrated the accumulation of the CnMV marker (14-3-3-GFP) and host reactive astrocytes (GFAP-positive cells) around cystic lesions, a new hallmark of *C. neoformans*-infected brains [4].

To further explore the pathological features of CnMVs in infected brains, confocal microscopy was performed to examine the fine structures around a cystic lesion. On day 16 post-infection, cystic lesions were examined (Fig. 7G) with CnMVs (14-3-3-GFP) distributed around the margin of a lesion. Multiple cysts are present near the center of the large cystic lesions (Fig. 7G, box 1). These cystic areas may represent areas containing lysed brain cells, edematous regions, or spaces filled with unstainable materials such as *C. neoformans* capsules. Some *C. neoformans* cells were observed inside the cystic (Supplement, Fig. S2). A common feature of these cystic regions was that they were surrounded by a high density of 14-3-3-GFP staining [Fig. 7G-1 and the 3D video in Supplement V(1)]. Some cystic lesions (Fig. 7G, box 2) showed intense green fluorescence around brain cortex cells and reactive astrocytes (red fluorescence) [Fig. 7G-2 and the 3D video in Supplement V(2)]. Interestingly, the 14-3-3-GFP was also observed inside the brain cells [Fig 7G-3 and the 3D video in Supplement V(3)] in the outskirts of the cystic lesions (Fig. 7B, Fig7G & 7G-3). Taken together, the high density of the CnMV marker 14-3-3-GFP inside and around the cystic lesions of infected brains suggests that CnMVs are closely associated with the pathogenesis of *C. neoformans* in infected brains.

## Discussion

Many types of cells secrete bioactive vesicles from the endosomal membrane system into their surrounding environments. These vesicles, often called exosomes or secreted MVs, play crucial roles in intercellular communication. *C. neoformans* are also found to produce such vesicles of 100–200 nm in diameter [17], [20,20]. In this study, we added two steps to the original protocol for CnMV isolation described by Rodrigues *et* *al.,* [20]. We first passed the culture supernatant through a 0.8-µm pore-size membrane filter to remove *C. neoformans* cells. After ultracentrifugation to pellet CnMVs, we performed sucrose gradient centrifugation to remove any potential contaminants (e.g., secreted proteins, capsule polysaccharides, cell debris, culture medium components). As described by others [20], we also detected acid phosphatase and luccase activities in the purified CnMVs.

The first indication in our study that CnMVs are involved in brain invasion of *C. neoformans* was from the experiments of *in* *vitro* blood-brain barrier model. Purified CnMVs derived from multiple strains of *C. neoformans* enhanced transcytosis *in* *vitro* (Fig. 2). These findings led us to believe that one essential step for *C. neoformans* pathogenesis involves the interaction of CnMVs with a target cell. Unlike *C. neoformans* cells [5], surface CnMVs did not colocalize with HBMEC CD44 (Fig. 3A), suggesting that the interaction between CnMVs and target cells is CD44-independent. However, this interaction elicited membrane raft activity, as evidenced by the presence of CD44, caveolin-1 in the lipid rafts, and also up-shifted the intracellular distribution of β-actin (Fig. 4A & B). These observations suggest that CnMVs exert profound effects on target cells. Since HBMEC CD44 is a receptor for *C. neoformans* adhesion [3]–[5], its migration to the lipid rafts (Fig. 4A) may be a mechanism of which CnMVs enhance *C. neoformans* transcytosis and adhesion (Figs. 2 & 5). Another intriguing observation is that CnMVs induced host cell fusions (Fig. 4C). Presumably, this fusion activity may assist in the adherence and internalization of CnMVs and/or *C. neoformans* into recipient cells (Fig. 3). This speculation remains to be demonstrated.

**Figure 8:**
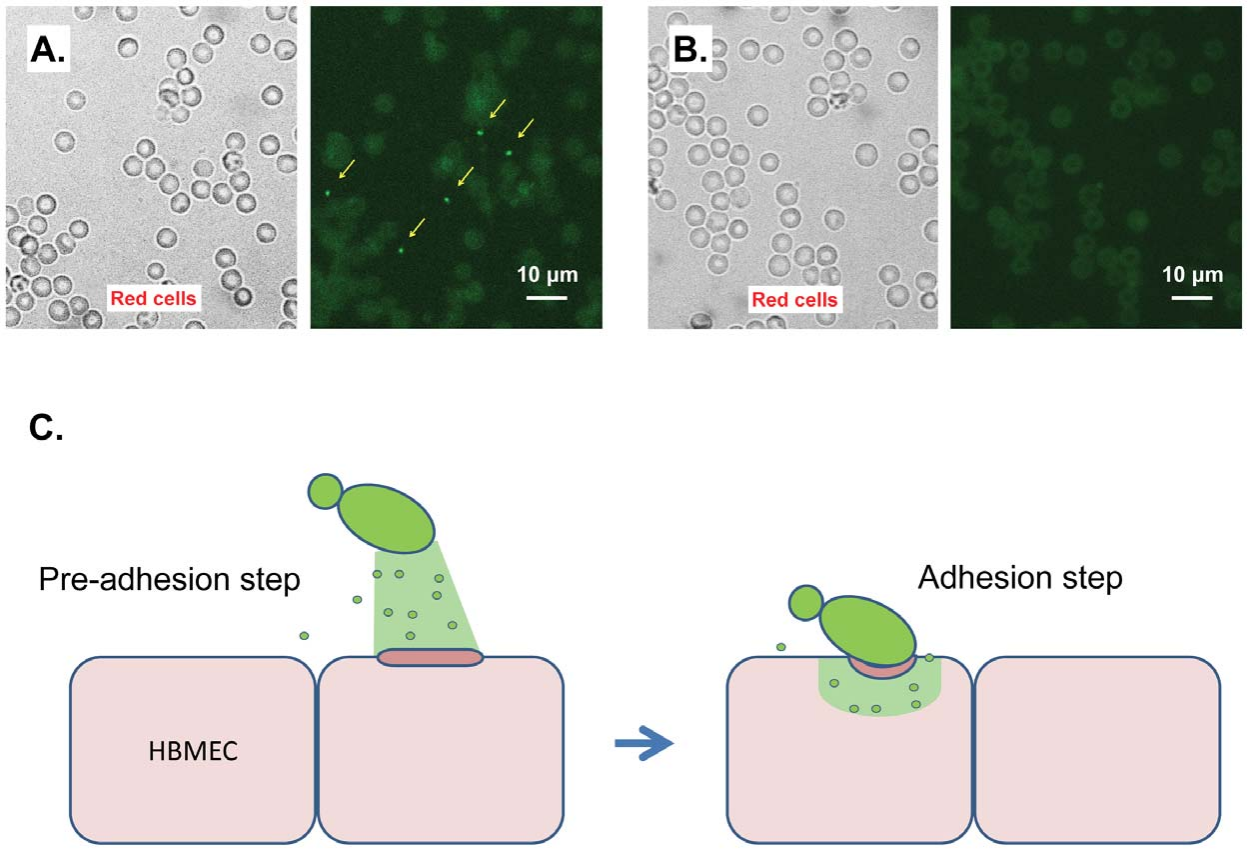
Detection of CnMV particles in infected mouse blood. Mice were intravenously injected with 5×10^6^ cryptococcal cells of either strain JBO2 (**A**) or strain C1186 (**B**). After 16 hr, a drop of blood was collected from either the eyes or the tail vein, and was plated onto the glass slide. Red blood cells were observed under the light microscope (left panel) and green CnMV particles (indicated by yellow arrows) could be observed in the immunofluorescence image of JBO2 sample (A: right panel). (**C**) A model of the putative role of disseminated CnMVs to facilitate *C. neoformans* traversal through the blood-brain barrier is shown. The middle section shows the monolayer of human brain microvascular endothelial cells (HBMEC) (light pink cells). *C. neoformans* cells (green) may continuously secret extracellular vesicles CnMVs into the blood stream. Intact CnMVs (green dots) and/or already-lysed components of the CnMV’s (*e.g*., green sector area) contact the HBMEC and exert some profound effects on the surface of HBMEC (dark pink bar), a pre-adhesion step. Subsequently, the active surface is facilitated for *C. neoformans* binding with the HBMEC, the adhesions step.

Heat-treated CnMVs did not have the ability to enhance the traversal of *C. neoforma*ns across the BBB (Fig. 2), suggesting that heat-labile factors are required for this function. Proteomic analysis showed that CnMVs contains more than 70 polypeptides [20], but which components are involved in adhesion to HBMECs (Fig. 5) is currently unknown. CnMVs may exert their effects on the surface of HBMECs or inside the host cells (Figs. 3–5). Although we have uncovered membrane activation and induction of cell fusion activities of CnMVs, there may be additional functions associated with CnMVs in the interaction between *C. neoformans* and its host cells (HBMECs and brain cells). It has been reported that CnMV particles are unstable in the presence of serum albumin [22] prompting the research team to investigate whether the CnMVs exert their functions in intact particles or in a lysed form. Therefore, we examined the blood smear slides from infected mice with cryptococcal cells from either JBO2 or C1186 strains (Fig. 8). Under the light microscope, the disc-shape red blood cells (RBCs) can easily be observed from both samples. However, the green CnMV particles can only be observed in the JBO2-infected mouse blood samples (Fig. 8A), and not from C1186 ones (Fig. 8B). Having examined twenty random fields, it is estimated that the ratio of RBC to green CnMV particles is roughly 1,000∶1. In Fig. 1B, numerous CnMV particles have accumulated around the cryptococcal cells. One simple hypothesis is that infected *C. neoformans* cells can continuously secret the CnMV particles into the blood stream, despite instability in the presence of serum albumin. The secreted CnMV components, in an intact or lysed form, may cultivate a pro-invasive microenvironment during its blood-borne-dissemination phase of cryptococcosis, a pre-adhesion step (Fig. 8C, left panel). Figure 5 supports our speculation that the preincubation of CnMV with HBMEC enhances cryptococcal cell binding to the host cells. Furthermore, in Fig. 4A, subpopulations of CD44 and caveolin-1 molecules redistribute to the membrane lipid rafts, a site of portal entry [3]–[5]. Subsequently, the active surface is facilitated for *C. neoformans* cells binding to the HBMEC, the adhesions step (Fig. 8C, right panel). Giving the complexity of CnMV components, this may just be a precursor in the CnMV triggered invasion mechanism.

One key component of this study was a sensitive CnMV marker that could be used *in* *vivo*. Using *C. neoformans*-derived 14-3-3-GFP as the marker for CnMVs, we showed that CnMVs congregated around the cystic lesions in infected brains (Fig. 7). Since it is known that *C. neoformans* cells begin to shed and replenish their capsule components as soon as they have invaded the brain [23] and that CnMVs are the major vesicles for the transport of capsule components [8], it was not surprising to find a substantial amount of 14-3-3-GFP inside infected cells (Fig. 7). The internalized 14-3-3-GFP was also found *in* *vitro* when HBMECs were incubated with *C. neoformans* JB02 cells (Fig. 3B). One possible explanation is that a *C. neoformans* cell invaded into a host cell and secreted its MVs internally. Similar vesiculation has been observed with other infectious pathogens. For example, *Leishmania* releases vesicles into the cytoplasm of infected macrophages [10], and MVs released by *Plasmodium ssp.* are found in the cytoplasm of infected erythrocytes. Therefore, it is plausible that MVs carry a number of virulence factors into targeted cells that cause detrimental effects.

Multiple cysts were found in the large cystic lesions in infected brains. Because 14-3-3-GFP was present at a high concentration in these areas, CnMVs may play a direct role in causing brain lesion. This observation is in agreement with the fact that MVs released from oral and gastric bacteria have important roles in periodontitis and gastric ulcers [14]. In response to infection or injuries, the activation of astrocytes can be characterized by their upregulation of intermediate filaments, such as GFAP, vimentin [24]. Indeed, one of the prominent features of the *C. neoformans*-infected brains is the GFAP-positive astrocytes surrounding the cystic lesions (Figure 7). Coincident with this observation, we have previously shown that *C. neoformans* capsule component GXM also surrounds the cystic lesion sites in a similar manner [4]. Colocalization of capsule components, CnMVs, and reactive astrocytes could be the hallmark of *C. neoformans* brain invasion [4]. CnMVs may contain several virulence factors to facilitate *C. neoformans* invasion. On the other hand, reactive astrocytes may play crucial roles in restricting the spread of invading yeast cells inside the infected brains [25]. The interplay between invading factors and host defensive mechanism(s) could be closely associated with the severity of meningoencephalitis.

Since CnMVs contain several known virulence factors, they are called “virulence bags” that facilitate infection [20]. The link between the various functions of CnMVs and *C. neoformans* pathogenesis needs to be further investigated. We found little to no apoptotic signals in the cystic lesions of the infected brains, but multiple types of hydrolytic enzyme activities were found in the purified MVs (data not shown). Given the plethora of roles associated with MVs in different organisms, it is conceivable that CnMVs have many yet to be identified pathogenic roles. We are currently fractionating the active components of CnMVs to understand how they interact with a host cell and the pathways they may activate during *C. neoformans* infection.

## Materials and Methods

### Ethics Statement

The animal study was performed in strict accordance with the recommendations in the Guide for the Care and Use of Laboratory Animals of the National Institutes of Health. Our protocols were approved by the Institutional Animal Care and Use Committee (IACUC) of The Saban Research Institute of CHLA (Permit number: A3276-01). All surgery was performed under anesthesia with ketamine and lidocaine, and all efforts were made to minimize suffering. All research involving human participants has been approved by the Institutional Review Board (IRB) of Children’s Hospital Los Angeles (CHLA). Human brain microvascular endothelial cells were isolated in accordance with the protocol approved by the CHLA Committee on Clinical Investigations (CCI), which is the IRB for Human Subjects at Saban Research Institute of CHLA. This protocol has been granted a waiver of informed or signed consent per 45 CFR 46.116(d) and a waiver of HIPAA authorization per the Privacy Rule (45 CFR Part 160 and Subparts A and E of Part 164). No minors/children participants were involved in our studies.

### Reagents and Materials

To construct the 14-3-3-GFP expression vector, the PCR primers 1433-N (CAAGATGTCTAACCGAGAAGACTCT) and 1433-C (GGAGGAGGTTGCCCCTGCGGCTTAGA CGAG) were used to amplify the approximately 0.8 kb *C. neoformans* 14-3-3 gene (Transcript: CNAG_05235T0). The resulting DNA fragment was cloned into pET28b-EGFP at BamHI and NotI sites to generate pET28b-1433-EGFP. The 1.6-kb 14-3-3-EGFP DNA fragment on pET28b-1433-EGFP was then amplified with PCR primers 69214 (pET upstream primer 5′-ATGCGTCCGGCGTAGA-3′; from Novagen, Inc.) and C-AscI (5′-CCTGGCGCGCCTTGTTAGCAGCCGGATCTCAG) and subcloned into pGEM-t-easy (Clontech) to generate the plasmid pGEM-MV11G. In this plasmid, the inserted 14-3-3-GFP DNA fragment contained a T7 tag and a 6×His tag at its N- and C-terminal ends, respectively (Fig. 1A). The DNA fragment containing the actin-1 promoter and URA5 selection marker was then inserted in front of the 14-3-3-GFP gene to generate the plasmid pACT1-MT15. The resulting plasmid was linearized with ApaI and electroporated into the strain B-4500FO2 for URA selection, and the resulting strain was designated JB02. The CellTracker dyes CMFDA (5-chloromethylfluorescein diacetate) (cat# C2925) and CMTPX: 4-(chloromethyl)6-(4(5)-amino-2-carboxyphenyl)-1,2,2,4,8,10,10, 11-octamethyl-1,2,10, 11-tetrahydro-dipyrido[3,2-b:2',3'-i] xanthylium (cat# C34552) were purchased from Invitrogen, Inc. They are used for staining in Figure 4 [26].

### Yeast Strains

*C. neoformans* strains JEC21, B-4500FO2, JB02, and C1186 were used. JEC21 [27] and B-4500FO2 [7], [28], [29] have been described. The C1186 and JB02 strains, which express GFP and 14-3-3-GFP, respectively, were derived from B-4500FO2. Yeast cells were grown aerobically at 30°C in 1% yeast extracts, 2% peptone, and 2% dextrose (YPD broth). Cells were harvested at the early log phase and washed with YPD, and the cell concentration was adjusted to an optical density = 1 ( ∼10^8^ cells/mL) prior to the experiments.

### *In vitro C. neoformans* Adhesion and Transcytosis Assays

HBMECs were isolated from human brain specimens as described previously and the *C. neoformans in* *vitro* adhesion assay and transcytosis assay was performed as previously described [30]. Briefly, for adhesion assay, HBMEC were grown in collagen-coated 24-well culture plates (Costar) until confluence. An inoculum of 10^6^ cryptococcus cells (strain B4500FO2) in 1 ml experimental medium was added (m.o.i. of ∼10) at 37°C for dose-dependent studies (0, 1, 2 and 4 µg CnMV) with or without 3 hr pre-incubation. After 3 hr, free yeast cells were removed from HBMEC by washing four times with experimental medium. Adherent yeast cells were retained by the HBMEC. Subsequently, HBMEC were lysed with 0.5%Triton, diluted and plated onto YPD agar plates and colonies were counted. For *in* *vitro* transcytosis assay, HBMECs were cultured on collagen-coated Transwell polycarbonate tissue-culture inserts with a pore diameter of 8 µm (Corning Costar) for 4 days. HBMECs were polarized and exhibited a trans-endothelial electrical resistance (TEER) of 250–300 μΩ per cm^2^, as measured with an Endohm volt/ohm meter (World Precision Instruments). One million *C. neoformans* cells were added to the upper chamber (total volume 500 µl) in the presence of purified CnMVs and incubated at 37°C. At different time intervals, samples (100 µl) were taken from the lower chamber and plated for colony forming unit (CFU) counting. Three measurements were made at each time point for each sample.

### Isolation of *C. neoformans*-derived Microvesicles (CnMVs)

CnMVs were isolated as described previously, with a slight modification [20]. The yeast cultures were centrifuged at 8,000 × *g* for 10 minutes. The supernatant was collected and filtered through a Millipore filter unit (0.8 µm pore size) to ensure no contamination of the preparation by cryptococcal cells. The crude CnMVs were collected from the supernatant by ultracentrifugation at 100,000 × *g* for 1h. The pellet was resuspended in PBS containing gentamicin and placed on a gradient of 0.5 mL of 0.5 M, 1 mL of 1.0 M, and 1 mL of 2.0 M sucrose solutions in buffer (20 mM HEPES, 150 mM NaCl, pH 7.4) in an SW51 tube and centrifuged at 100,000 × *g* for 2 hours. After centrifugation, the purified CnMVs were harvested from the interface between the 1.0 and 2.0 M sucrose solutions. The quantity of CnMVs is based on the amount of proteins, determined by the Bradford assay (Bio-Rad Laboratories, Hercules, CA).

### Preparation of Membrane Lipid Rafts from HBMECs

The Caveolae/Rafts Isolation kit was purchased from Sigma Chemical Co. (cat. # CS0750). For each sample, HBMECs were seeded and grown in a 60-mm Petri dish for 2 days. On the day of experiment, the cells were incubated with 0, 1 2, or 4 µg purified CnMVs for 2 hours, and the cell extracts were subjected to density gradient centrifugation. Nine fractions (0.5 mL each) were collected from the top to the bottom of SW40 centrifuge tubes. Forty microliters from each fraction were used for protein blots using antibodies against CD44 (Santa Cruz Biotech, sc-7964, 1∶300 dilution), caveolin-1 (Cell Signaling Technology, #3251, 1∶500), β-actin (Santa Cruv Biotech, sc-8432, 1∶400 dilution), and vimentin (Santa Cruz Biotech, sc-6260, 1∶1,000 dilution). An anti-mouse-HRP conjugate (1∶2,000 dilution) was also used.

### *In vivo* Animal Studies

Groups of 3–5 female C57LB/6 mice were injected with a total of 10^6^ cryptococcal cells and 0, 50, or 100 µg purified CnMVs via the lateral tail vein. After 24 hours, the brains were removed, washed with 2 mL PBS, homogenized and plated in triplicate on YPD plates for determination of *C. neoformans* CFU (Fig. 6A). A published method for isolation of *C. neoformans* from CSF was followed with slight modifications [31]. Briefly, groups of 5–8 female C57LB/6 mice were injected with a total of 10^6^ cryptococcal cells and 0, 50, or 100 µg purified CnMVs or 50 µg heat-inactivated CnMVs via the lateral tail vein. After 24 hours, each animal was perfused with 20 mL PBS, and the skull was opened. The brain was removed and washed with 500 µL PBS. Concurrently, the cranial cavity was washed with 100 µL PBS four times, after which the washing solutions were combined. After centrifugation, the pellet was resuspended in 50 µL PBS and designated as the CSF fraction.

### Immunofluorescence Microscopy

Samples for immunofluorescence microscopy were prepared as described previously [5], [32]. Briefly, HBMECs (∼5×10^4^ cells) were seeded on one coverslip 24 hours prior to an experiment. HBMECs were washed four times with PBS, fixed with 2% formaldehyde/PBS (v:v) for 30 min, blocked with 5% milk/PBS for 30 min, and incubated with an anti-CD44 monoclonal antibody (Santa Cruz Biotech, sc-7297), an anti-GFP antibody (Abcam, ab290), and an anti-rabbit antibody-FITC conjugate overnight at 4°C. The coverslips were washed 4 times with PBS before one drop of Vectashield mounting solution with DAPI was added (Vector Laboratory, H-120) to seal the coverslips onto the slides. For the brain sections, mouse brains were fixed with 4% formaldehyde overnight and the processed for paraffin embedding. A microtome was used to cut the paraffin block into 5–10 µm thick sections. CnMVs in the brain sections were stained with an anti-GFP antibody or an anti-GFAP (glial fibrillary acidic protein) monoclonal antibody (Cell Signaling Technology), and an FITC-conjugated (green) or rhodamine-conjugated (red) secondary antibody. Fluorescence images were acquired with an LSM 710 confocal system mounted on an AxioObserver.Z1 inverted microscope equipped with a 63x/1.4 Plan-APOCHROMAT oil-immersion objective lens (Carl Zeiss Microimaging).

### Statistical Analysis

Considering both the global and individual features of our data, statistical analysis was carried out by the combination of ANOVA (a global test) and the Newman-Keuls test (a post-hoc test). Analysis of variance (ANOVA) was used for statistical analysis of the data obtained in this study. The dependent variable was the percent of cells or the CFU, whereas the independent fixed factors were the treatments (different strains, different amounts of MVs, etc.). Raw data were entered into Microsoft Excel files and automatically converted to statistical packages. ANOVA and co-variates were followed by a multiple comparison test such as the Newmann-Keuls test (a post-hoc test) to determine the statistical significance between the control and treatment groups. *P*<0.05 was considered significant.

## Supporting Information

Figure S1 **Distribution of CnMVs in an infected mouse brain.** Mice were infected with a total of 10^6^ yeast cells by lateral tail vein injection. Small cystic lesions were observed within 24 hours after infection as seen in split confocal images at sections of 3.94 µm, 7.39 µm, and 10.83 µm from the top (A) and a complete set of images from top to bottom (B). Host astrocytes (red), *C. neoformans* secreted microvesicles (green), and brain nuclear DNA (blue) were shown. Green yeast cells were more difficult to distinguish due to the high intensity of 14-3-3-GFP florescence in this area. It appeared that in a cystic lesion, the yeast cells continuously secreted their extracellular microvesicles (stained green), which elicited the activation of the host astrocytes (GFAP-positive, red) to the infection site.
(TIF)

Figure S2 **CnMVs in large cystic lesions.** Brain sections of the post-16 day infection were displayed at sections of 8.86 µm (left) and 12.80 µm (Right) from the top. Host astrocytes (red), *C. neoformans* secreted microvesicles (green), and brain nuclear DNA (blue) were shown. The number and size of cystic lesions increased over time during the infection. Increased lesions to the brain cortex were observed as the infection became more severe. As seen in a representative confocal microscopic image, many cystic regions surrounding a high intensity of green staining were observed (left panel). Two side-view images, reconstituted by the ScanLine program, confirmed that these cystic regions were open, unstained spaces. Occasionally, green stains representing *C. neoformans* cells were seen in some cystic regions (right panel). A large population of reactive astrocytes (red) was also observed at the same section.
(TIF)

Video S1 **Three-dimensional image was reconstructed by the confocal microscope-associated Zen program from** **Figure 7G** **-1, showing high density of 14-3-3-GFP signals surrounding the unstained regions of the cystic lesion.**
(MOV)

Video S2 **Three-dimensional image was reconstructed by the confocal microscope-associated Zen program from** **Figure 7G** **-2, showing significant 14-3-3-GFP staining and a reactive astrocyte in the cystic lesion.**
(MOV)

Video S3 **Three-dimensional image was reconstructed by the confocal microscope-associated Zen program from** **Figure 7G** **-3, showing a brain cell filled with green staining.**
(MOV)
