# Supplementary figures and images for: Cryptococcus neoformans-Derived Microvesicles Enhance the Pathogenesis of Fungal Brain Infection
Source: PLoS One. 2012 Nov 7;7(11):e48570. doi: 10.1371/journal.pone.0048570 (PMC3492498; doi:10.1371/journal.pone.0048570)

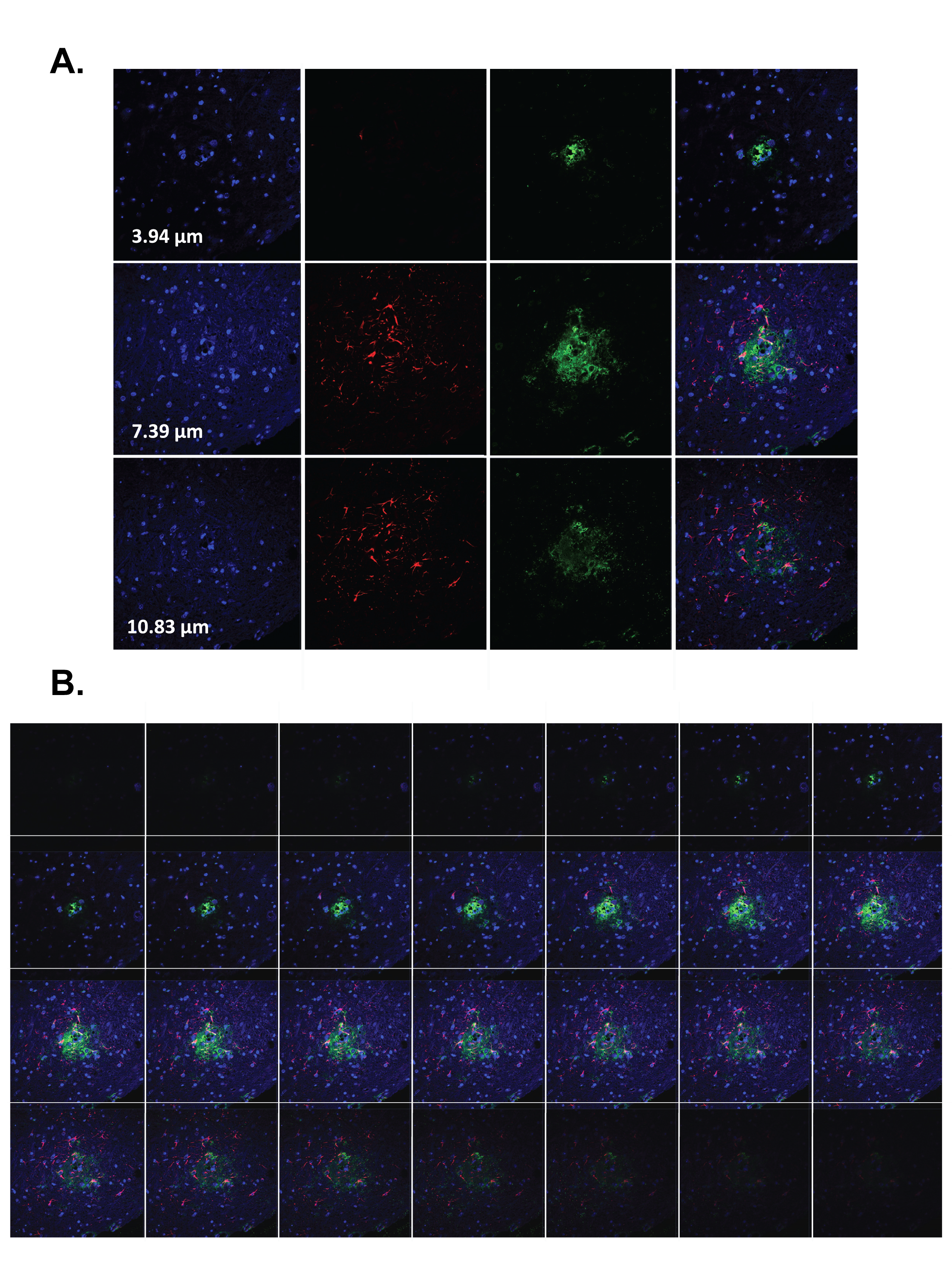

Supplement: Figure S1 — Distribution of CnMVs in an infected mouse brain. Mice were infected with a total of 106 yeast cells by lateral tail vein injection. Small cystic lesions were observed within 24 hours after infection as seen in split confocal images at sections of 3.94 µm, 7.39 µm, and 10.83 µm from the top (A) and a complete set of images from top to bottom (B). Host astrocytes (red), C. neoformans secreted microvesicles (green), and brain nuclear DNA (blue) were shown. Green yeast cells were more difficult to distinguish due to the high intensity of 14-3-3-GFP florescence in this area. It appeared that in a cystic lesion, the yeast cells continuously secreted their extracellular microvesicles (stained green), which elicited the activation of the host astrocytes (GFAP-positive, red) to the infection site. (TIF) [file pone.0048570.s001.tif]

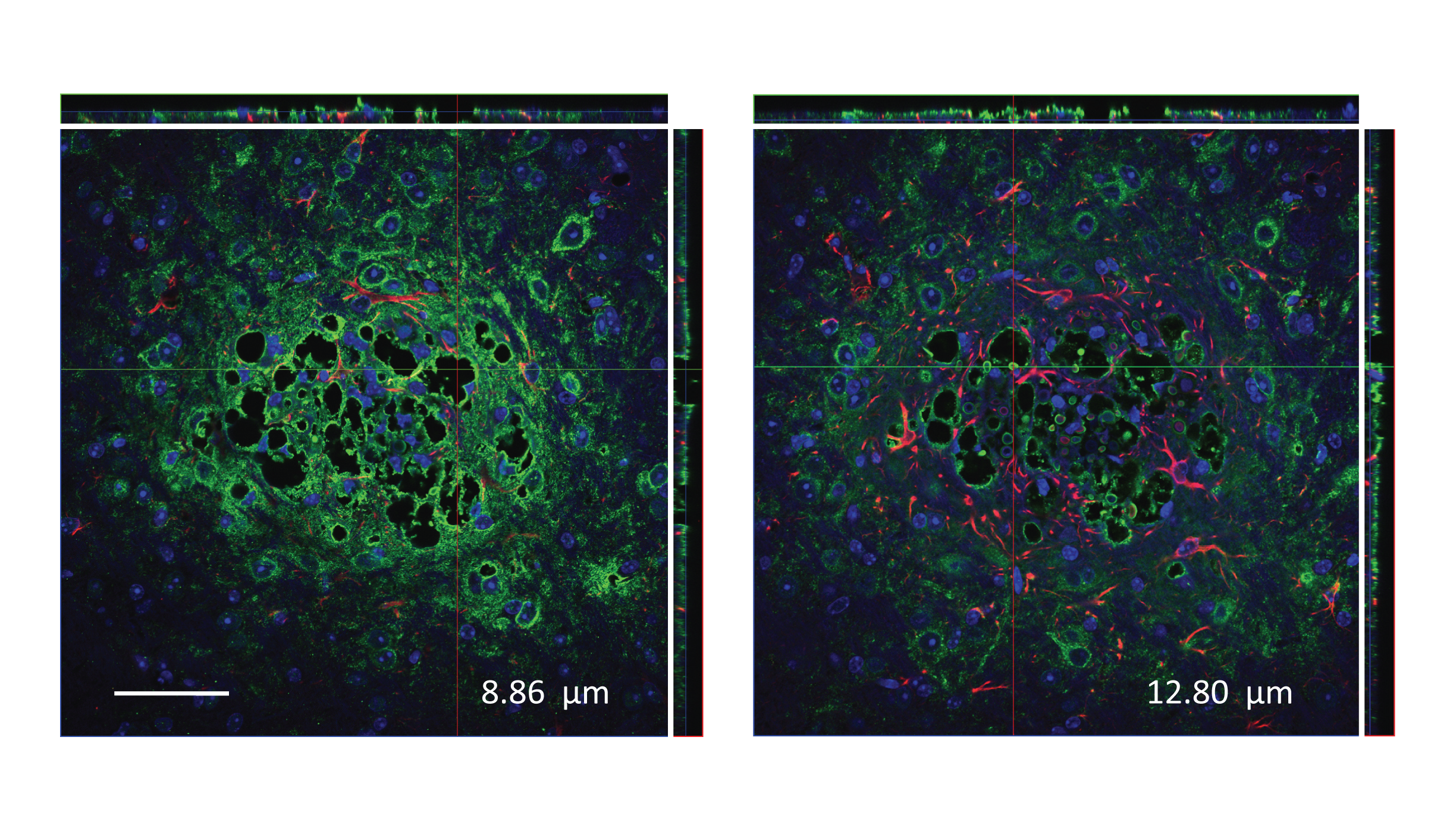

Supplement: Figure S2 — CnMVs in large cystic lesions. Brain sections of the post-16 day infection were displayed at sections of 8.86 µm (left) and 12.80 µm (Right) from the top. Host astrocytes (red), C. neoformans secreted microvesicles (green), and brain nuclear DNA (blue) were shown. The number and size of cystic lesions increased over time during the infection. Increased lesions to the brain cortex were observed as the infection became more severe. As seen in a representative confocal microscopic image, many cystic regions surrounding a high intensity of green staining were observed (left panel). Two side-view images, reconstituted by the ScanLine program, confirmed that these cystic regions were open, unstained spaces. Occasionally, green stains representing C. neoformans cells were seen in some cystic regions (right panel). A large population of reactive astrocytes (red) was also observed at the same section. (TIF) [file pone.0048570.s002.TIF]
